# Dietary Phytoestrogen Intake is Inversely Associated with Hypertension in a Cohort of Adults Living in the Mediterranean Area

**DOI:** 10.3390/molecules23020368

**Published:** 2018-02-09

**Authors:** Justyna Godos, Sonia Bergante, Angela Satriano, Francesca Romana Pluchinotta, Marina Marranzano

**Affiliations:** 1 Department of Medical and Surgical Sciences and Advanced Technologies “G.F. Ingrassia”, University of Catania, 95125 Catania, Italy; marranz@unict.it; 2 Department of Biomedical and Biotechnological Sciences, University of Catania, 95123 Catania, Italy; 3 IRCCS Policlinico San Donato, 20097 San Donato Milanese (MI), Italy; sonia.bergante@unimi.it (S.B.); angela.satriano@gmail.com (A.S.); francescaromana.pluchinotta@cardio.chboston.org (F.R.P.)

**Keywords:** phytoestrogen, lignan, isoflavone, blood pressure, hypertension, CVD, meditteranean cohort

## Abstract

Background: Dietary polyphenols, including phytoestrogens are abundantly present in a balanced diet. Evidence for their role in preventing non-communicable diseases is emerging. Methods: We examined the association between estimated habitual intakes of dietary phytoestrogens and hypertension in a cohort study. The baseline data included 1936 men and women aged 18 years and older. Intakes of total phytoestrogens, isoflavones, and lignans were calculated from validated food frequency questionnaire. Data on the polyphenols content in foods were retrieved from the Phenol-Explorer database. Results: Individuals in the highest quartile of dietary phytoestrogens intake were less likely to be hypertensive (OR: 0.66, 95% CI: 0.44–0.98); moreover, the association showed a significant decreasing trend. Isoflavones and lignans were not associated with lower odds of hypertension; however, some individual compounds, such as biochanin A and pinoresinol showed an independent inverse association with hypertension. Conclusions: Dietary phytoestrogens are associated with lower likelihood of hypertension in adults living in the Mediterranean area. Future studies are needed to confirm the present findings (i.e., prospective cohort studies) and to better understand the mechanisms underlying such associations.

## 1. Introduction

Dietary polyphenols are the focus of major attention due to their potential beneficial effects on human health [1,2]. Among the others, phytoestrogens are a group of molecules with weak, yet clinically relevant, estrogen-like activity. Among the most studied groups of phytoestrogens, isoflavones and lignans have been demonstrated to be the most consumed in human diets [3]. Phytoestrogens bind to the estrogen receptors (ERs) and can act both as agonists and antagonists [4]. Specifically, the dualistic mode of action of isoflavones depends on the fact that they can activate ER-alpha, promoting cell proliferation, and ER-beta, promoting apoptosis [4]. Lignans have been reported to weakly bind to ER-beta and stimulate estrogen-stimulated growth [5]. Generally, phytoestrogens have been studied for their ability to regulate cell proliferation/apoptosis and in relation to cancer risk/protection. However, recent evidence suggests that phytoestrogen-rich foods may play a role in modulating cardiovascular risk, providing the rationale to pay broader attention to these molecules for prevention of non-communicable diseases [6].

Phytoestrogens naturally occur in soy products, which are traditionally consumed in Asian countries; soy intake ranges approximately from 70 to 150 g/d in Asian diets, corresponding to a daily intake of isoflavones of about 30 to 60 mg. However, phytoestrogens are also contained in certain legumes (i.e., beans and peas) and nuts as well as fruits, vegetables, grains and seeds [7]. Thus, their consumption, even though at lower amounts, has also been registered in US and European countries. Consumption of isoflavones has been estimated to be around 2.5 mg/d in both US and European countries, while average lignans intake in Europe is 1.5 mg/d [8,9]. However, dietary intake of phytoestrogens has been reported to be very low in populations living in the Mediterranean area: on average, taking into account countries such as Greece, Spain, and Italy, intake of both isoflavones and lignans ranges from 10 μg/d to up to approximately 1 mg/d [9,10]. However, another investigation conducted in Southern Italy showed higher average intake of both isoflavones (4 mg/d) and lignans (3 mg/d) [11]. Several studies have been conducted on Asian cohorts exploring the effects of soy/isoflavones and lignans on human health, but studies including individuals living in Mediterranean countries are scarce [12,13,14]. Even though soy products tend to be infrequently consumed in Mediterranean countries, there are other food sources of lignans that are common components of a traditional Mediterranean dietary pattern. Overall, the effect on hypertension of habitual low-dose consumption of phytoestrogens is unknown. Therefore, the aim of this study was to explore the association between dietary phytoestrogens intake and blood pressure (BP) in a cohort of southern Italian adults.

## 2. Results

Baseline characteristics of participants in the Mediterranean healthy eating, aging, and lifestyle (MEAL) study by quartiles of dietary phytoestrogens intake are described in Table 1. 

Among individuals in the highest category of phytoestrogen intake, there was a higher percentage of females. Participants with higher total phytoestrogen intake had lower occupational levels. Regarding lifestyle behaviors, there was a higher prevalence of non-smokers, low physical active and regular alcohol drinkers among high phytoestrogen consumers compared to lower ones. With increasing intake of phytoestrogens, a higher intake of sodium and potassium was also observed. However, some differences occurred when analysis was stratified by sex: in the highest quartile of phytoestrogen intake men had lower educational level while women were younger (Supplementary Table S1).

A total number of 976 individuals were hypertensive or taking medication to reduce high BP. Participants with no hypertension had an appreciably higher mean intake of total phytoestrogens compared to hypertensive individuals (8.33 mg/d vs. 5.19 mg/d, respectively). To this difference mostly contributed mean amount of isoflavones intake between non-hypertensive and hypertensive individuals (5.40 mg/d vs. 2.60 mg/d, respectively; *p* < 0.001), while mean intake of lignans was rather similar, despite yet significantly different between the two groups (2.92 mg/d vs. 2.59 mg/d, respectively; *p* = 0.005). When the total phytoestrogens intake between non-hypertensive and hypertensive individuals was stratified by sex, the difference was larger for women (9.72 mg/d vs. 5.68 mg/d, respectively; *p* < 0.001) than for men (5.78 mg/d vs. 4.65 mg/d, respectively; *p* = 0.076), with similar pattern of difference for isoflavones and lignans (data not shown).

**Figure 1:**
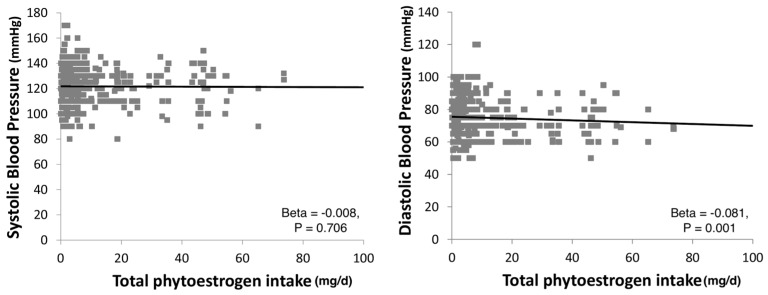
Association between dietary phytoestrogen intake and systolic and diastolic blood pressure (BP).

The relation between total phytoestrogens and BP levels showed a significant linear association with diastolic but not systolic blood pressure (SBP, Figure 1).

The association between quartiles of total and individual phytoestrogen intake and hypertension is reported in Table 2. 

In the multivariable-adjusted analysis individuals in the highest quartile of total phytoestrogen intake were less likely to be hypertensive (OR: 0.66, 95% CI: 0.44–0.98); moreover, the association showed a significant decreasing trend (Table 2). Among the main classes of phytoestrogens, neither isoflavones nor lignans were associated with lower odds of hypertension. However, some individual compounds, such as biochanin A (OR: 0.63, 95% CI: 0.41–0.96) and pinoresinol (OR: 0.59, 95% CI: 0.39–0.89) showed an independent association with hypertension (Table 2). In the stratified analysis by sex (Supplementary Table S2), only dietary intake of pinoresinol in men was significantly inversely associated hypertension (OR: 0.47, 95% CI: 0.23–0.93).

## 3. Discussion

The present study showed that individuals reporting higher intake of total phytoestrogens were less likely to be hypertensive. Interestingly, significant results were found only when considering total phytoestrogen intake, while no association was found for isoflavone and lignan intake individually. This finding suggests that phytoestrogens may share a common mechanism of action leading to the prevention of hypertension and that the necessary threshold to reach a clinically relevant effect was not reached when considering an individual group of molecules. However, consumption of both isoflavones and lignans was not significantly associated with hypertension and a larger sample with stronger statistical power may have had detected significant results also for both classes of phytoestrogens evaluated individually.

Results of this study are in line with some existing evidence related to phytoestrogens and cardiovascular health. Findings from randomized controlled trials provide the strongest evidence of causal effect but they are generally limited by the short duration and might be affected by the usual consumption of the examined compounds if diet is not controlled: for instance, trials conducted on Asian population with high habitual average phytoestrogen intake showed different results than trials conducted on Western population. However, meta-analyses of randomized controlled trials on soy supplementation showed a significant reduction in BP, despite results were not univocal: indeed, subgroup analyses showed more consistent evidence when considering hypertensive, but not normotensive individuals [15] with no dose-response relationship [16]. Meta-analyses of randomized controlled trials on lignan-rich foods, such as sesame [17], black cumin [18], and flaxseed [19], have provided evidence of a significant effect in reducing BP. Nevertheless, some randomized-controlled trials showed null or scanty significant results [20]. It is noteworthy to underline that most of the intervention trials provided supplementation of lignans at doses that might have been not sufficient to observe any relevant clinical effect. Observational studies may provide a more realistic picture of the habitual consumption of foods in a population, but they are affected by a several biases and potential confounding factors. The potential beneficial effect of several foods on hypertension has been pointed out [21,22,23]: summary evidence syntheses showed substantial beneficial effects of soy [24], nuts [25,26], legumes [27,28], coffee [29,30] on cardiovascular-related outcomes. However, previous observational studies specifically investigating phytoestrogens showed mixed results on their potential association with hypertension. A study conducted on European postmenopausal women showed that dietary isoflavones intake was not associated with several parameters of endothelial function [31]. However, the authors explored the aforementioned associations considering the exposure as continuous variables, which may affect the results due to the influence of extreme data estimates compared to categorization (i.e., quantiles). Another study conducted on Polish adults showed no association between isoflavones and lignans intake and features of metabolic syndrome, including impaired BP [32]; A prospective evaluation of the same cohort in relation to risk of hypertension substantially confirmed previous results [28].

As aforementioned in the introduction, it is noteworthy to consider that dietary sources of phytoestrogens in Mediterranean cohorts may differ from European, American, and Asian cohorts. Previous results from the MEAL cohort showed that the major food contributors to isoflavones intake were legumes (beans), nuts and seeds rather than soy foods, which were consumed in much lower amounts than the former, while major contributors of lignans were citrus fruits (including red orange), garlic, olive oil and bread [33]. These foods are characteristic of a traditional Mediterranean dietary pattern [34], which has been shown to be associated with potential benefits toward BP and inversely associated with hypertension in the MEAL cohort [35]. Interestingly, another study also showed that increasing isoflavones and lignans intake was associated with higher adherence to the Mediterranean diet, suggesting that these polyphenol groups may exert or mediate the potential beneficial effects of this dietary pattern on BP and endothelial function [36].

From a mechanistic point of view, several hypotheses have been proposed to explain the potential protective effects of dietary phytoestrogens on endothelial function. Primarily, phytoestrogens are able to emulate physiological responses equivalent to those evoked by physiological estrogens (i.e., endothelium-dependent vasodilatation) [23]. Isoflavones are thought to influence flow-mediated dilation through effects on the acute response cell-signaling pathways that increase nitric oxide (NO) production [37]. Moreover, isoflavones significantly reduce circulating adhesion molecules, such as intercellular adhesion molecule-1, (ICAM-1) vascular cell adhesion molecule-1 (VCAM-1), and E-selectin, which in turn reduce platelet activation, and may explain the antithrombotic and platelet aggregation inhibition effects of isoflavones [38]. Lignans have potent antioxidant properties and have been shown to decrease in vivo lipid peroxidation, which in turn may prevent endothelium damage and atheromatous plaque formation [39]. Another mechanism responsible for BP lowering effect of lignans is a NO-mediated vasorelaxation action [40]. Lignans have been demonstrated to directly decrease endothelin-1 synthesis and increase NO production, which in turn may lead to arterial vasodilation and to reduction in BP [40]. Moreover, the BP lowering properties of lignans may be independent of their anti-oxidative effect, acting through reduction of 20-hydroxyeicosatetraenoic (20-HETE) levels, a metabolite of arachidonic acid which has been hypothesized to play a key role in the pathogenesis of hypertension in humans [41].

To the best of our knowledge, this is the first study investigating the association between dietary phytoestrogens and hypertension in a Mediterranean cohort. However, the findings should be considered in light of some limitations. First, the cross-sectional design of the study may provide insights on the association between variables but causal relation cannot be concluded. Second, data on dietary phytoestrogens was derived from an FFQ (Food Frequency Questionnaire), which may be preferred to rank rather than to be used to derive the absolute amount of compounds. However, foods containing high amounts of phytoestrogens are most likely to be consumed periodically, rather than on a daily basis, thus an FFQ would be a preferable choice, compared to 24-h recalls, to estimate the dietary content of phytoestrogens. Moreover, used questionnaires provided a valid estimation of the foods that might contribute to the intake of isoflavones and lignans (i.e., fruits, vegetables, legumes, coffee, tea, nuts, and grain products). Another limitation of exploring dietary intake of phytoestrogens is the potential confounding effect of other compounds that have demonstrated BP-lowering effects, such as fiber and omega-3 polyunsaturated fatty acids, high SFA and Na intake [42,43]. Finally, several factors in addition to diet, such as intestinal microflora, smoking, antibiotics, and obesity may affect bioavailability and circulating phytoestrogen levels in the body; thus, additional studies on intermediary biomarkers of phytoestrogen consumption are warranted to confirm the results obtained in this study.

## 4. Materials and Methods

### 4.1. Study Design and Population

The MEAL study is an observational study designed in order to investigate the relationship between nutritional and lifestyle habits characterizing the classical Mediterranean area and non-communicable diseases. The baseline data included a random sample of 2044 men and women aged 18 or more years old, and randomly selected in the main districts of the city of Catania, southern Italy. The enrollment and data collection was performed between the years 2014 and 2015 through the selection among the lists of registered patients of a pool of general practitioners. Details of the study protocol are published elsewhere [44]. All participants were informed about the aims of the study and provided a written informed consent. All the study procedures were carried out in accordance with the Declaration of Helsinki (1989) of the World Medical Association. The study protocol has been reviewed and approved by the concerning ethical committee.

### 4.2. Data Collection

An electronic data collection was performed by face-to-face assisted personal interview using tablet computers. In order to visualize the response options, participants were provided with a paper copy of the questionnaire. However, final answers were registered directly by the interviewer. The demographic data included gender, age at recruitment, highest educational degree achieved, occupation (specifies the character of the most important employment during the year before the investigation) or last occupation before retirement, and marital status; educational status was categorized as (i) low (primary/secondary); (ii) medium (high school); and (iii) high (university). Occupational status was categorized as (i) unemployed; (ii) low (unskilled workers); (iii) medium (partially skilled workers); and (iv) high (skilled workers). Physical activity status was evaluated using International Physical Activity Questionnaires (IPAQ) [45], which included a set of questionnaires (5 domains) investigating the time spent being physically active in the last 7 days. Based on the IPAQ guidelines, final score allows categorizing physical activity level as (i) low; (ii) moderate; and (iii) high. Smoking status was categorized as (i) non-smoker; (ii) ex-smoker; and (iii) current smoker. Alcohol consumption was categorized as (i) none; (ii) moderate drinker (0.1–12 g/d) and (iii) regular drinker (>12 g/d).

### 4.3. Dietary Assessment

The dietary assessment has been performed by the administration of two food frequency questionnaires (a long and a short version) that have been previously tested for validity and reliability for the Sicilian population [46,47]. The identification of the food intake, the energy content as well as the macro- and micro-nutrients intake were obtained through comparison with food composition tables of the Research Center for Foods and Nutrition [48]. Intake of seasonal foods referred to consumption during the period in which the food was available and then adjusted by its proportional intake in one year. FFQs with unreliable intakes (<1000 or >6000 kcal/d) were excluded from the analyses (*n* = 107) leaving a total of 1936 individuals included in the analysis. 

### 4.4. Estimation of Polyphenol Intake

The process of the estimation of phytoestrogen intake has been previously described in detail [49]. Briefly, data on the polyphenol content in foods were retrieved from the Phenol-Explorer database (www.phenol-explorer.eu) [50,51]. A new version of the Phenol-Explorer database containing data on the effects of cooking and food processing on polyphenol contents was used whenever possible in order to apply polyphenol-specific retention factors [51]. Foods that contained no polyphenols were excluded from the calculation, leaving a total of 75 items included in the analyses. Food weight loss or gain during cooking was corrected using yield factors [52]. The average food consumption was calculated (in g or mL) by following the standard portion sizes used in the study and then converted in 24-h intake. Finally, a search was carried out in the Phenol-Explorer database to retrieve mean content values for all polyphenols contained in the selected foods. Next, phytoestrogen intake from each food was calculated by multiplying the content of each phytoestrogen class by the daily consumption of each food. The total phytoestrogen intake was considered as the sum of total isoflavones and lignans; individual phytoestrogens, including the isoflavones daidzein, genistein and biochanin A, and the lignans lariciresinol, matairesinol, pinoresinol, and seicoisolariciresinol were also calculated. Finally, pyhtoestrogens intake was adjusted for total energy intake (kcal/d) using the residual method [53]. 

### 4.5. Anthropometric Measurements and Outcome Ascertainment

Anthropometric measurements have been collected following standard procedures [54]. Arterial blood pressure (BP) was measured in sitting position and at least 5 min at rest at the end of the physical examination. Because of the possibility of differences in BP measurement, the measurements were taken three times at the right arm relaxed and well supported by a table, with an angle of 45° from the trunk. A mean of the last two measurements was considered for inclusion in the database. Information from measurements was integrated with general practitioners computerized records, as patients are diagnosed with disease by a specialist in order to obtain drug reimbursement. Patients have been considered hypertensive when average systolic/diastolic BP levels were higher or equal to 140/90 mm Hg, taking anti-hypertensive medications, or being previously diagnosed of hypertension.

### 4.6. Statistical Analysis

Frequencies are expressed as absolute numbers and percentages; continuous variables are expressed as means and standard deviations. Individuals were divided into quartiles of dietary phytoestrogens intake and distribution of background characteristics were compared between groups. Differences were tested with Chi-square test for categorical variables, ANOVA for continuous variables distributed normally, and Kruskall-Wallis test for variables distributed not normally. A linear logistic regression analysis was performed to test the relation between total phytoestrogen intake and systolic and diastolic blood pressure (DBP). Age- and energy-adjusted multivariate logistic regression models were used to test association between variables of exposure (including total phytoestrogens and individual subclasses and specific compound intake) and having hypertension; additional multivariate models adjusted for all other background characteristics (age, sex, educational and occupational status, smoking and alcohol drinking habits, physical activity level, sodium and potassium intake) were also performed to test whether the observed associations were independent from the aforementioned potential confounding factors. All reported *p*-values were based on two-sided tests and compared to a significance level of 5%. SPSS 17 (SPSS Inc., Chicago, IL, USA) software was used for all the statistical calculations.

## 5. Conclusions

In conclusions, dietary phytoestrogens are associated with lower likelihood of hypertension in adults living in the Mediterranean area. Future studies are needed to confirm the present findings (i.e., prospective cohort studies) and better understand the mechanisms underlying such associations.

## Figures and Tables

**Table 1:** Baseline characteristics by quartiles of dietary phytoestrogens intake in the Mediterranean healthy eating, aging, and lifestyle (MEAL) study sample (*n* = 1936).

	Total Phytoestrogens Intake				*p*
	Q1 (Median = 258.56)	Q2 (Median = 432.43)	Q3 (Median = 604.97)	Q4 (Median = 1054.63)	
Age (years), mean (SD)	47.9 (18.7)	49.4 (17.2)	49.5 (17.6)	46.7 (16.7)	0.040
Sex, *n* (%)					0.011
Male	190 (23.6)	224 (27.9)	217 (27.0)	173 (21.5)	
Female	272 (24)	271 (23.9)	276 (24.4)	313 (27.7)	
Educational level, *n* (%)					0.434
Low	161 (23.1)	168 (24.1)	188 (27.0)	180 (25.8)	
Medium	168 (23.3)	195 (27.1)	188 (26.1)	169 (23.5)	
High	133 (25.6)	132 (25.4)	117 (22.5)	137 (26.4)	
Occupational level, *n* (%)					0.001
Unemployed	109 (23.6)	85 (18.4)	121 (26.2)	146 (31.7)	
Low	51 (19.2)	57 (21.4)	88 (33.1)	70 (26.3)	
Medium	109 (24.8)	119 (27.0)	113 (25.7)	99 (22.5)	
High	131 (26.7)	125 (25.5)	131 (27.3)	104 (25.3)	
Smoking status, *n* (%)					0.014
Non smoker	270 (22.6)	296 (24.8)	301 (25.2)	328 (27.4)	
Ex-smoker	121 (26.0)	121 (26.0)	111 (23.9)	112 (24.1)	
Current smoker	71 (25.7)	78 (28.3)	81 (29.3)	46 (16.7)	
Physical activity, *n* (%)					<0.001
Low	89 (27.1)	55 (16.7)	83 (25.2)	102 (31.0)	
Medium	221 (25.8)	238 (27.8)	175 (20.4)	222 (25.9)	
High	97 (17.9)	159 (29.3)	165 (30.4)	122 (22.5)	
Alcohol consumption, *n* (%)					<0.001
No	97 (25.9)	95 (25.3)	84 (22.4)	99 (26.4)	
Moderate (<12 g/d)	311 (25.8)	303 (25.1)	334 (27.7)	258 (21.4)	
Regular (≥12 g/d)	54 (15.2)	97 (27.2)	75 (21.1)	130 (36.5)	
Na (microg/d), mean ± SD	2724.6 ± 1155.7	2869.9 ± 1045.8	2870.8 ± 1060.4	2947.8 ± 1147.3	0.021
K (microg/d), mean ± SD	2920.4 ± 897.0	3269.6 ± 941.2	3915.5 ± 1068.3	4589.9 ± 1806.8	<0.001
Systolic blood pressure (mmHg), mean ± SD	122.1 ± 12.9	121.3 ± 12.4	121.4 ± 12.2	121.5 ± 13.8	0.723
Diastolic blood pressure (mmHg), mean ± SD	76.1 ± 9.9	75.7 ± 9.9	75.1 ± 10.3	73.4 ± 11.2	<0.001

**Table 2:** Association between quartiles of dietary phytoestrogens intake (total and main classes) and hypertension.

	OR (95% CI)			
Polyphenols (mg/d)	Q1	Q2	Q3	Q4
Total phytoestrogens, median (range)	0.89 (0.20, 1.23)	1.77 (1.24, 2.33)	3.61 (2.33, 5.41)	10.21 (5.42, 101.72)
No. of cases	247	260	257	212
Model ^a^	1	0.85 (0.63, 1.14)	0.79 (0.58, 1.07)	0.68 (0.49, 0.96)
Model ^b^	1	0.87 (0.61, 1.26)	0.69 (0.47, 1.00)	0.66 (0.44, 0.98)
Isoflavones, median (range)	0.01 (0.00, 0.01)	0.03 (0.01, 0.04)	0.07 (0.05, 0.08)	5.53 (0.09, 92.98)
No. of cases	240	267	265	204
Model ^a^	1	0.97 (0.72, 1.31)	0.87 (0.64, 1.17)	0.96 (0.69, 1.34)
Model ^b^	1	0.81 (0.56, 1.15)	0.94 (0.65, 1.35)	0.93 (0.64, 1.36)
Daidzein, median (range)	0.01 (0.00, 0.01)	0.02 (0.01, 0.02)	0.04 (0.03, 0.05)	0.14 (0.05, 6.49)
Model ^a^	1	1.14 (0.84, 1.55)	1.03 (0.76, 1.40)	0.94 (0.68, 1.29)
Model ^b^	1	0.95 (0.66, 1.36)	1.05 (0.73, 1.52)	1.03 (0.70, 1.51)
Genistein, median (range)	0.00 (0.00, 0.00)	0.01 (0.00, 0.01)	0.02 (0.01, 0.02)	0.14 (0.02, 7.77)
Model ^a^	1	1.23 (0.91, 1.65)	1.04 (0.76, 1.42)	0.99 (0.72, 1.36)
Model ^b^	1	1.05 (0.73, 1.49)	1.08 (0.73, 1.58)	1.07 (0.73, 1.57)
Biochanin A, median (range)	0.00 (0.00, 0.00)	0.36 (0.10, 0.50)	0.95 (0.70, 1.70)	2.50 (1.80, 34.3)
Model ^a^	1	0.99 (0.72, 1.35)	1.24 (0.91, 1.69)	0.63 (0.46, 0.87)
Model ^b^	1	0.99 (0.65, 1.48)	0.99 (0.66, 1.50)	0.63 (0.41, 0.96)
Lignans, median (range)	0.71 (0.00, 1.07)	1.41 (1.07, 1.99)	2.46 (2.00, 3.62)	5.41 (3.63, 23.56)
No. of cases	225	232	275	244
Model ^a^	1	0.66 (0.49, 0.89)	1.14 (0.83, 1.56)	0.77 (0.55, 1.09)
Model ^b^	1	0.59 (0.41, 0.86)	0.88 (0.61, 1.27)	0.71 (0.47, 1.08)
Lariciresinol, median (range)	0.25 (0.00, 0.48)	0.69 (0.48, 1.06)	1.23 (1.06, 2.00)	3.11 (2.01, 13.98)
Model ^a^	1	0.65 (0.48, 0.88)	1.03 (0.76, 1.40)	0.79 (0.56, 1.10)
Model ^b^	1	0.60 (0.41, 0.86)	0.84 (0.58, 1.21)	0.69 (0.46, 1.03)
Matairesinol, median (range)	0.00 (0.00, 0.01)	0.01 (0.01, 0.02)	0.02 (0.02, 0.04)	0.06 (0.04, 0.30)
Model ^a^	1	1.00 (0.74, 1.35)	1.47 (1.07, 2.01)	1.03 (0.73, 1.44)
Model ^b^	1	0.95 (0.65, 1.37)	1.08 (0.74, 1.58)	0.91 (0.60, 1.38)
Pinoresinol, median (range)	0.31 (0.00, 0.41)	0.52 (0.41, 0.70)	0.87 (0.70, 1.24)	1.75 (1.25, 7.30)
Model ^a^	1	0.62 (0.46, 0.85)	0.94 (0.68, 1.29)	0.65 (0.46, 0.92)
Model ^b^	1	0.58 (0.40, 0.84)	0.79 (0.54, 1.16)	0.59 (0.39, 0.89)
Secoisolariciresinol, median (range)	0.03 (0.00, 0.05)	0.06 (0.05, 0.08)	0.11 (0.08, 0.14)	0.21 (0.14, 0.90)
Model ^a^	1	0.78 (0.58, 1.06)	1.11 (0.80, 1.53)	0.80 (0.56, 1.14)
Model ^b^	1	0.73 (0.51, 1.06)	0.88 (0.60, 1.30)	0.69 (0.44, 1.06)

^a^ Model adjusted for age (years, continuous), energy intake (kcal/d, continuous), sodium and potassium intake; ^b^ Model adjusted for age (years, continuous), energy intake (kcal/d, continuous), smoking status (smokers, ex-smokers, non-smokers), alcohol consumption (0 g/d, <12 g/d, ≥12 g/d), physical activity level (low, medium, high), educational level (low, medium, high), occupational level (unemployed, low, medium, high), sodium and potassium intake.

## Supplementary Materials

The following are available online at http://www.mdpi.com/1420-3049/23/2/368/s1, Supplementary Table S1: Background characteristics by quartiles of dietary phytoestrogens intake in the MEAL study sample separately for men and women, Supplementary Table S2: Association between quartiles of dietary phytoestrogens intake (total and main classes) and hypertension in men and women.
