# Supplementary material for: Dietary Phytoestrogen Intake is Inversely Associated with Hypertension in a Cohort of Adults Living in the Mediterranean Area
Source: Molecules. 2018 Feb 9;23(2):368. doi: 10.3390/molecules23020368 (PMC6017940; doi:10.3390/molecules23020368)
Supplement: Supplementary file 1 [file molecules-23-00368-s001.pdf]

Supplementary Table 1. Background characteristics by quartiles of dietary phytoestrogens intake in the MEAL study sample separately for men and women.

	Total phytoestrogens intake									
	Men (n= 804)					Women (n = 1132)				
	Q1 (median = 257.51)	Q2 (median = 435.15)	Q3 (median = 615.12)	Q4 (median = 1042.52)	P	Q1 (median = 259.66)	Q2 (median = 432.43)	Q3 (median = 599.16)	Q4 (median = 1055.22)	P
Educational level, n (%)					0.214					0.134
Low	51 (20.5)	68 (27.3)	67 (26.9)	63 (25.3)		110 (24.6)	100 (22.3)	121 (27.0)	117 (26.1)	
Medium	85 (26.0)	99 (30.3)	87 (26.6)	56 (17.1)		83 (21.1)	96 (24.4)	101 (25.7)	113 (28.8)	
High	54 (23.7)	57 (25.0)	63 (27.6)	54 (23.7)		79 (27.1)	75 (25.8)	54 (18.6)	83 (28.5)	
Occupational level, n (%)					0.042					0.065
Unemployed	14 (20.0)	11 (15.7)	23 (32.9)	22 (31.4)		95 (24.3)	74 (18.9)	98 (25.1)	124 (31.7)	
Low	31 (22.0)	31 (22.0)	49 (34.8)	30 (21.3)		20 (16.0)	26 (20.8)	39 (31.2)	40 (32.0)	
Medium	52 (22.7)	67 (29.3)	58 (25.3)	52 (22.7)		57 (27.0)	52 (24.6)	55 (26.1)	47 (22.3)	
High	70 (28.8)	60 (24.7)	74 (30.5)	39 (16.0)		61 (24.6)	65 (26.2)	57 (23.0)	65 (26.2)	
Smoking status, n (%)					0.386					0.200
Non smoker	98 (22.2)	120 (27.1)	116 (26.2)	108 (24.4)		172 (22.8)	176 (23.4)	185 (24.6)	220 (29.2)	
Ex smoker	48 (25.8)	50 (26.9)	51 (27.4)	37 (19.9)		73 (26.2)	71 (25.4)	60 (21.5)	75 (56.9)	
Current smoker	44 (25.0	54 (30.7)	50 (28.4)	28 (15.9)		27 (27.0)	24 (24.0)	31 (31.0)	18 (18.0)	
Physical activity, n (%)					<0.001					0.056
Low	32 (31.4)	15 (14.7)	18 (17.6)	37 (36.3)		57 (25.1)	40 (17.6)	65 (28.6)	65 (28.6)	
Medium	97 (28.7	106 (31.4)	61 (18.0)	74 (21.9)		124 (23.9)	132 (25.5)	114 (22.0)	148 (28.6)	
High	48 (16.8)	85 (29.8)	108 (37.9)	44 (15.4)		49 (19.0)	74 (28.7)	57 (22.1)	78 (30.2)	
Alcohol consumption, n (%)					<0.001					0.003
No	34 (26.2)	40 (30.8)	27 (20.8)	29 (22.3)		63 (25.8)	55 (22.2)	57 (23.4)	69 (28.3)	
Moderate (<12 g/d)	135 (26.3)	141 (27.5)	150 (29.2)	87 (17.0)		176 (25.4)	162 (23.4)	184 (26.6)	171 (24.7)	
Regular (≥12 g/d)	21 (13.0)	43 (26.7)	40 (24.8)	57 (35.4)		33 (16.9)	54 (27.7)	35 (17.9)	73 (37.4)	
Age group, mean (SD)	47.34 (19.66)	50.36 (17.31)	48.24 (16.49)	47.71 (16.91)	0.294	48.42 (18.14)	48.76 (17.24)	50.61 (18.39)	46.28 (16.67)	0.030

Na, mean (SD)	2914.87 (1353.70)	2804.82 (1067.79)	2910.89 (1071.51)	2872.13 (1130.85)	0.741	2591.69 (975.16)	2923.7 (1026.16)	2839.79 (1052.73)	2990.62 (1157.25)	<0.001
K, mean (SD)	2930.31 (938.07)	3241.38 (848.24)	4061.96 (981.13)	4429.11 (1614.08)	<0.001	2913.60 (868.95)	3292.93 (1012.53)	3802.09 (1120.00)	4682.53 (1898.02)	<0.001

Supplementary Table 2. Association between quartiles of dietary phytoestrogens intake (total and main classes) and hypertension in men and women.

	Men, OR (95% CI)				Women, OR (95% CI)			
	Q1	Q2	Q3	Q4	Q1	Q2	Q3	Q4
Total phytoestrogens								
No. of cases	113	127	132	90	134	133	125	122
Model [a]	1	0.72 (0.47, 1.11)	0.91 (0.57, 1.46)	0.72 (0.42, 1.21)	1	0.98 (0.64, 1.49)	0.58 (0.37, 0.91)	0.71 (0.44, 1.13)
Model [b]	1	0.80 (0.47, 1.37)	0.73 (0.41, 1.30)	0.65 (0.34, 1.25)	1	1.10 (0.64, 1.87)	0.59 (0.34, 1.01)	0.73 (0.42, 1.27)
Isoflavones								
No. of cases	96	132	137	97	144	135	128	107
Model [a]	1	1.22 (0.79, 1.90)	1.09 (0.70, 1.70)	1.36 (0.81, 2.28)	1	0.75 (0.49, 1.13)	0.68 (0.45, 1.04)	0.73 (0.46, 1.15)
Model [b]	1	1.06 (0.62, 1.80)	1.04 (0.59, 1.83)	1.46 (0.77, 2.77)	1	0.61 (0.36, 1.03)	0.88 (0.54, 1.46)	0.74 (0.45, 1.24)
Daidzein								
Model [a]	1	1.17 (0.75, 1.83)	1.17 (0.74, 1.84)	1.11 (0.67, 1.84)	1	1.07 (0.70, 1.63)	0.89 (0.57, 1.37)	0.83 (0.53, 1.30)
Model [b]	1	0.89 (0.52, 1.52)	0.91 (0.51, 1.61)	1.36 (0.71, 2.59)	1	1.04 (0.62, 1.75)	1.26 (0.75, 2.12)	0.98 (0.58, 1.66)
Genistein								
Model [a]	1	1.29 (0.83, 2.00)	1.28 (0.80, 2.03)	1.13 (0.69, 1.85)	1	1.10 (0.72, 1.66)	0.83 (0.53, 1.29)	0.88 (0.57, 1.38)
Model [b]	1	1.01 (0.60, 1.72)	1.02 (0.57, 1.82)	1.34 (0.71, 2.51)	1	1.06 (0.64, 1.77)	1.16 (0.68, 1.98)	1.02 (0.61, 1.72)
Biochanin A								
Model [a]	1	0.88 (0.53, 1.47)	1.36 (0.83, 2.23)	0.59 (0.36, 0.96)	1	1.01 (0.66, 1.53)	0.97 (0.63, 1.48)	0.50 (0.32, 0.80)
Model [b]	1	0.76 (0.38, 1.50)	1.05 (0.54, 2.04)	0.61 (0.31, 1.20)	1	1.28 (0.74, 2.21)	1.00 (0.57, 1.77)	0.61 (0.33, 1.12)
Lignans								
No. of cases	103	105	147	107	122	127	128	137
Model [a]	1	0.53 (0.33, 0.85)	1.23 (0.77, 1.96)	0.70 (0.41, 1.20)	1	0.80 (0.52, 1.22)	0.85 (0.54, 1.34)	0.78 (0.49, 1.25)
Model [b]	1	0.48 (0.27, 0.86)	0.83 (0.48, 1.45)	0.63 (0.32, 1.22)	1	0.80 (0.47, 1.36)	0.82 (0.48, 1.41)	0.76 (0.43, 1.33)
Lariciresinol								
Model [a]	1	0.71 (0.45, 1.11)	1.48 (0.94, 2.34)	0.82 (0.49, 1.38)	1	0.59 (0.39, 0.90)	0.59 (0.37, 0.92)	0.71 (0.44, 1.13)

Model [b]	1	0.65 (0.37, 1.14)	1.02 (0.59, 1.77)	0.74 (0.38, 1.41)	1	0.60 (0.35, 1.02)	0.65 (0.38, 1.10)	0.62 (0.36, 1.09)
Matairesinol								
Model [a]	1	0.98 (0.62, 1.54)	2.05 (1.28, 3.30)	1.08 (0.64, 1.82)	1	0.95 (0.62, 1.45)	0.81 (0.51, 1.28)	0.88 (0.54, 1.41)
Model [b]	1	0.83 (0.48, 1.45)	1.29 (0.74, 2.30)	0.86 (0.44, 1.66)	1	1.12 (0.66, 1.91)	0.89 (0.51, 1.54)	0.93 (0.52, 1.65)
Pinoresinol								
Model [a]	1	0.48 (0.30, 0.76)	0.87 (0.54, 1.41)	0.59 (0.35, 1.01)	1	0.77 (0.50, 1.17)	0.84 (0.54, 1.32)	0.64 (0.40, 1.02)
Model [b]	1	0.40 (0.22, 0.72)	0.60 (0.33, 1.08)	0.47 (0.23, 0.93)	1	0.90 (0.53, 1.53)	0.88 (0.51, 1.51)	0.63 (0.35, 1.13)
Secoisolariciresinol								
Model [a]	1	0.86 (0.55, 1.34)	1.12 (0.70, 1.80)	0.76 (0.44, 1.31)	1	0.70 (0.45, 1.07)	0.92 (0.58, 1.47)	0.81 (0.50, 1.34)
Model [b]	1	0.77 (0.44, 1.35)	0.73 (0.41, 1.29)	0.58 (0.29, 1.15)	1	0.80 (0.46, 1.36)	0.99 (0.56, 1.75)	0.78 (0.43, 1.43)

[a] Model adjusted for age (years, continuous), energy intake (kcal/d, continuous), sodium and potassium intake.

[b] Model adjusted for age (years, continuous), energy intake (kcal/d, continuous), smoking status (smokers, ex-smokers, non-smokers), alcohol consumption (0 g/d,<12 g/d, ≥12 g/d), physical activity level (low, medium, high), educational level (low, medium, high), occupational level (unemployed, low, medium, high), sodium and potassium intake.
